# The emergence of Kawasaki disease in India and China

**DOI:** 10.21542/gcsp.2017.21

**Published:** 2017-10-31

**Authors:** Fuyong Jiao, Ankur Kumar Jindal, Vignesh Pandiarajan, Raju Khubchandani, Nutan Kamath, Tapas Sabui, Rakesh Mondal, Priyankar Pal, Surjit Singh

**Affiliations:** 1 Children’s Hospital, Shaanxi Provincial People’s Hospital of Xian, Jiaotong Univeristy, China; 2 Allergy Immunology Unit, Department of Pediatrics, Advanced Pediatrics Centre, PGIMER, Chandigarh, India; 3 Department of Pediatrics, Jaslok Hospital, Mumbai, India; 4 Department of Pediatrics, Kasturba Medical College, Mangalore, India; 5 Department of Pediatrics, RG Kar Medical College, Kolkata, India; 6 Institute of Post Graduate Medical Education and Research, Kolkata, India; 7 Institute of Child Health, Kolkata, India

## Abstract

Kawasaki disease (KD) is recognized as a leading cause of acquired heart disease in children in developed countries. Although global in distribution, Japan records the highest incidence of KD in the world. Epidemiological reports from the two most populous countries in the world, namely China and India, indicate that KD is now being increasingly recognized. Whether this increased reporting is due to increased ascertainment, or is due to a true increase in incidence, remains a matter of conjecture. The diagnosis and management of KD in developing countries is a challenging proposition. In this review we highlight some of the difficulties faced by physicians in managing children with KD in resource-constrained settings.

## Introduction

Kawasaki disease (KD) is recognized as a leading cause of acquired heart disease in children in developed countries, having replaced acute rheumatic fever as the most common cause. Over 60 countries across the world have reported KD to date, with robust epidemiological data available only from Japan, Taiwan, Korea, USA, UK, and Australia^1,2^. However, it should be noted that varying methodologies have been used to study the epidemiology of KD in different countries. These include passive surveillance from past hospital records, case registries, and active surveillance. Over the last two decades, awareness of KD amongst pediatricians in the world’s two most populous countries - China and India - has increased significantly and this condition is being reported increasingly frequently. There is anecdotal evidence that KD may soon replace rheumatic fever to become the commonest cause of acquired heart disease in children in both these countries.

## Epidemiology of KD

Japan records the highest number of cases of KD in the world, with ∼12,000 new cases being identified each year^3^. An updated estimation of the national incidence rate for KD is 322 per 100,000 children <5 years^4^. Approximately 85% of affected children with KD are younger than 5 years, although Indian data suggest that almost a third of patients are older. The male to female ratio is approximately 1.5:1^1^. In India there has been a steady increase in the number of cases diagnosed to have KD since the mid-1990s^5^. At many centres in India, KD has surpassed Henoch Schonlein purpura as the most common childhood vasculitic disorder^6^.

Unique epidemiological patterns have been identified in different regions. For instance, incidence rates in Far East Asian countries like Japan, Korea, and Taiwan are well above 50/100,000 children <5 years and, for reasons that are not clear, the incidence rates have continued to rise over the last 2 decades^2^. However, incidence in the United States, Canada, Australia and the European Union is around 4-25/100,000 children <5 years and these rates have reached a plateau^2^.

Data from the two most populous countries in the world, namely China and India, indicate that KD is now being increasingly recognized^2^. Whether this increased reporting is due to increased recognition of KD, or is a true increase in incidence, remains a matter of conjecture. Robust nationwide data are not available from either of these countries.

### Epidemiology in China

Regional epidemiological studies are available from China that show varying incidence in different geographic regions^7,8^. However, the overall trend in incidence of KD appears to be on the rise^2^. A questionnaire-based study data from Beijing (2000–04)showed an increase in incidence from 40.9 to 55.1 per 100,000 children <5 years^7^. Similar trends were noted in Shanghai where the incidence increased from 27.3 per 100,000 (1998–2002) to 46.3 per 100,000 in 2007^9^. Mean incidence in Sichuan Province was documented to be 7.1 per 100,000 children <5 years^10^. Data from Hong Kong also showed an increase in incidence from 26 per 100,000 children <5 years in 1994 to 39 per 100,000 in 2000 and to 74 per 100,000 in 2011^2^.

### Epidemiology in India

Before 1990, there were only 3 published reports of KD from India and the first report was published by Taneja et al. in 1977. However, over the last 20 years, several centres in India have started reporting KD^11–13^. A PubMed search with the terms “KD AND India” shows 161 citations with only 8 before 2000. The first two published case series came from Thiruvananthapuram in South India and Chandigarh in North India in the year 1997^14,15^. Since then, KD has been recognized in almost all parts of the country. A telephonic and questionnaire-based survey among physicians in India showed that KD is definitely being increasingly recognized in India. This could be either due to an actual rise in number of cases or due to increased awareness amongst pediatricians^16^.

Many senior pediatricians in India are of the opinion that KD was virtually non-existent in the country before the 1980s. India has witnessed a considerable surge in industrialization and economic productivity since the early 1990s. This coincided with the rise in number of cases of KD being reported from various parts of the country. Moreover, Kerala, which is one of the most developed states in India, also reports the largest number of cases of KD. Many physicians and pediatricians are of the view that rise in KD coincided with the fall of incidence of diarrhea and better vaccination coverage rates^16^.

A hospital based study from Chandigarh, North India showed an increase in incidence of KD from 0.51 per 100,000 children <15 years of age in the year 1994 to 4.5 per 100,000 children <15 years of age in the year 2007^5^. Peak incidence was noted in of October with a nadir in February. These incidence rates were speculated to be underestimates because of unrecognized and missed cases in the community due to lack of awareness amongst physicians and pediatricians. A follow-up study on the same pattern found that the mean incidence of KD at Chandigarh during the period 2009–2014 was 5.35/100,000 children <5 years^17^.

## Issues in diagnosis of KD in developing countries

1. For many physicians in developing countries like China and India, where the burden of infectious disease is high, KD is still not commonly included in the differential diagnosis of children presenting with fever. Antimicrobials are commonly prescribed for febrile episodes, and if the fever does not subside, a broader spectrum antimicrobial is often substituted. Some of the cardinal manifestations of KD (e.g., fever, rash and lymphadenitis) are also seen in many paediatric infectious diseases and it is not surprising that KD gets overlooked in such a milieu. Paediatricians in developing countries need to be sensitized about KD.
2. The presence of associated viral infections may not rule out the possibility of KD as both may co-exist in the same patient.
3. Although KD is now being diagnosed in most parts of China and India, there is no shortage of sceptics who refute the diagnosis of KD^18^. If diagnosis of KD is proffered by a paediatrician and the parents seek a second opinion, it is not unusual to encounter situations where this possibility is negated completely. As a result the patients may not get appropriate treatment for KD even when the condition has been correctly diagnosed.
4. In areas where awareness about KD is still not optimal, the treating paediatricians may not be aware that the clinical features of KD are transient and may change from day to day. As the fever persists, parents often seek multiple consultations. As a result many of the clinical findings that were present during the first few days of the disease may have subsided by the time the child is seen by another paediatrician - this often results in missed diagnosis.
5. Although KD is now being commonly recognized by paediatricians, it is not a part of undergraduate teaching curriculum in most medical schools. It is also not given the importance that it deserves in post-graduate medical curricula. Many adult physicians and cardiologists are still unaware of the devastating coronary sequelae associated with this condition.

## Issues related to echocardiography in KD

1. It is important to understand that echocardiography performed during the first 7 days of fever can never rule out a diagnosis of KD. If abnormalities are detected, it may confirm a clinical suspicion of KD. In developing countries like China and India, the echocardiography for KD is usually carried out by an adult cardiologist or a technologist (especially in China), who may not have enough expertise to perform echocardiographic evaluation of the coronary arteries in young infants. This is due to a paucity of trained paediatric cardiologists in developing countries.
2. Further, it is not unusual for the cardiologist to refute a diagnosis of KD on the basis of normal echocardiographic findings. In such situations when there is a difference of opinion between the paediatrician and the cardiologist, it becomes very difficult for the parents to decide on a reasonable course of action. Many parents may, in fact, opt not to go for treatment even when they have been told that there is no doubt about the diagnosis of KD. The cost of treatment may also be a factor in this decision.
3. The interpretation of echocardiographic findings is another issue that has not been adequately addressed in developing countries such as China and India. Many centres are still using the Japanese criteria for defining coronary artery abnormalities. Although these criteria are useful for initial screening, for more accurate assessments it is mandatory to use Z scores (internal dimension of the coronary artery expressed as the standard deviation from the mean normalized for body surface area)^19^. Though some centres in India (including Chandigarh, Kolkata and Mumbai) have starting using Z scores for assessment of coronary artery diameters, this is still not a routine practice in many parts of the country. The situation in China is similar. As a result there could be problems in interpreting the echocardiographic findings in a given child. Findings on echocardiographic examinations carried out at different centres are often not comparable and this adds to the diagnostic dilemma.

## Role of non-invasive coronary angiography in KD

Newer imaging modalities, such as dual source 128-slice computed tomography (DSCT) coronary angiography and magnetic resonance coronary angiography, are not being used frequently in developing countries as facilities are limited. At Chandigarh we have been using DSCT coronary angiography since 2014. This technique has been found to be extremely useful in the follow-up of patients with KD. Abnormalities in distal segments of coronary arteries that were missed on echocardiography could be picked up by this technique. Similarly, abnormalities in the circumflex coronary artery that may be difficult to identify on echocardiography are picked up by this imaging technique. However, the expertise to carry out and interpret the findings of CT coronary angiography is a significant barrier for using this imaging modality more widely in developing countries^20–22^.

## The dilemma of ‘incomplete’ and ‘atypical’ KD

1. Though the terms ‘incomplete’ and ‘atypical’ KD have, at times, been used interchangeably, they represent clinically distinct conditions. A child is considered to have incomplete KD when there are fewer than 4 clinical features in the presence of fever^1^. “Atypical KD” should be used in the presence of an unusual or odd manifestation of KD (e.g., nephritis or central nervous system complication)^23–26^. Incomplete KD is especially difficult to recognize as the diagnosis can be very difficult even for an experienced physician^27^. Young infants often have incomplete forms of KD and, in developing countries where infectious diseases are very common, may often be misdiagnosed to have viral exanthemata^28^. Incomplete KD is, by no means, a mild form of KD. On the contrary, such children may have significant coronary artery sequelae as the diagnosis and treatment are often delayed^29^.
2. All paediatricians need to be familiar with some of the pathognomonic clinical findings of KD that are not emphasized in the American Heart Association (AHA) diagnostic criteria^30^. These include reactivation of the Bacillus Calmette–Guérin (BCG) injection site^29^, sterile pyuria^31,32^, perineal peeling, arthritis, myocarditis, and hydrops of gall bladder. In difficult cases it is these clinical findings that may help the paediatrician in arriving at a diagnosis. Pro-BNP (Brain natriuretic peptide) estimation has recently been evaluated for inclusion in the diagnostic criteria of KD^33,34^. However, this laboratory investigation needs further validation for use in clinical practice.

## Consequences of a missed diagnosis of KD

No paediatrician can afford to miss a diagnosis of KD as the consequences can be grave^35,36^. The diagnosis of KD has to be considered upfront in all children where the fever persists for 5 days or more, even in the context of a developing country. The risk of coronary artery involvement is nearly 1 in 4 cases (25%) if left untreated^37^. Once giant coronary aneurysms develop, they are almost always irreversible.^1,37^ This risk can be significantly curtailed to less than 3% if intravenous immunoglobulin is administered within the first 10–12 days of fever. Missed KD in childhood can result in long-term coronary sequelae and affected patients can present in young adulthood with coronary ischemia, myocarditis, myocardial infarction, arrhythmias or sudden death. Thus the early diagnosis and prompt management of KD in childhood can have important implications for long-term cardiac morbidity. Adult cardiologists need to be familiar with these sequelae. At present, most adult cardiologists, especially in developing countries, may not be conversant with the ravages of missed KD in childhood. A recent publication from India demonstrates that this is slowly changing^38^.

## Issues in the management of KD

1. The treatment of KD involves use of intravenous immunoglobulin (IVIG)^1^. It is an expensive product and many families in developing countries may not be in a position to afford this treatment. IVIG is available free of cost in some regions (e.g., Shanghai in China; New Delhi, West Bengal and Kerala in India).
2. Although availability of IVIG is a major challenge in some developing countries, this is not a problem in either China or India. With the increased ascertainment of cases of KD in these countries, one can expect a major challenge to the existing health-care systems.

## Other forms of therapy for KD

In situations where administration of IVIG is not feasible for reasons of availability or affordability, one can consider alternative modes of therapy like glucocorticoids as a desperate measure. However, it should be understood that head to head trials comparing IVIG and glucocorticoids have never been carried out and, considering the proven efficacy of IVIG in KD, it may not be ethically possible to conduct such trials in the future. Such trials may only be feasible in countries where access to IVIG is extremely difficult.

## Outcomes of KD

The mortality reported in our cohort of children with KD from Chandigarh is 0.8%^39^. This is significantly higher than mortality figures of ≤0.04% reported from developed countries^35,39^. This increased mortality is largely attributed to delays in diagnosis and institution of therapy, especially in infants. In addition, the burden of coronary artery disease that may emerge if KD patients remain under-diagnosed and untreated will be a significant contributory factor to the long-term cardiac morbidity and mortality in these patients. Thus, KD is by no means a one-time disease of childhood. It has significant public health importance, especially for developing countries like China and India where the diagnosis of KD is often missed or delayed.

Epidemiologic data suggest that by 2030, in USA the estimated prevalence of KD would be 1 in 1,600 individuals^40^; in Taiwan the figure would be 1 in 700 individuals^41^. It is difficult to develop similar projections for developing countries because of lack of accurate epidemiologic data on KD in these countries. However, it is very likely that the consequences of missed KD in childhood in these countries will impact the public health resources in the years to come.

## References

[1] Newburger JW, Takahashi M, Gerber M (2004). Diagnosis, treatment, and long-term management of Kawasaki disease: A statement for health professionals from the Committee on Rheumatic Fever, Endocarditis and Kawasaki Disease, Council on Cardiovascular Disease in the Young, American Heart Association. Circulation.

[2] Singh S, Vignesh P, Burgner D (2015). The epidemiology of Kawasaki disease: A global update. Arch Dis Child.

[3] Makino N, Nakamura Y, Yashiro M (2015). Descriptive epidemiology of Kawasaki disease in Japan, 2011–2012: From the results of the 22nd nationwide survey. J Epidemiol.

[4] Sano T, Makino N, Aoyama Y, Ae R, Kojo T, Kotani K, Nakamura Y, Yanagawa H (2016). Temporal and geographical clustering of Kawasaki disease in Japan: 2007–2012. Pediatr Int.

[5] Singh S, Aulakh R, Bhalla AK (2011). Is Kawasaki disease incidence rising in Chandigarh, North India?. Arch Dis Child.

[6] Singh S, Kawasaki T (2009). Kawasaki disease - An Indian perspective. Indian Pediatr.

[7] Du Z-D, Zhao D, Du J (2007). Epidemiologic study on Kawasaki disease in Beijing from 2000 through 2004. Pediatr Infect Dis J.

[8] Chen J-J, Ma X-J, Liu F (2016). Epidemiologic features of Kawasaki Disease in Shanghai from 2008 through 2012. Pediatr Infect Dis J.

[9] Ma X, Yu C, Huang M (2010). Epidemiologic features of Kawasaki disease in Shanghai from 2003 through 2007. Chin Med J (Engl).

[10] Li X, Li X, Li H, Xu M, Zhou M (2008). Epidemiological survey of Kawasaki disease in Sichuan province of China. J Trop Pediatr.

[11] Singh S, Kawasaki T (2016). Kawasaki disease in India, lessons learnt over the last 20 years. Indian Pediatr.

[12] Kamath N, Shenoy R (2007). Kawasaki syndrome in coastal India. Indian Pediatr.

[13] Sarkar S, Mondal R, Nandi M, Ghosh A (2011). Trends of childhood vasculitides in eastern India. Indian Pediatr.

[14] Narayanan SN, Krishnaveni null, Sabarinathan K (1997). Kawasaki disease. Indian Pediatr.

[15] Singh S, Kumar L, Trehan A, Marwaha RK (1997). Kawasaki disease at Chandigarh. Indian Pediatr.

[16] Kushner HI, Macnee RP, Burns JC (2009). Kawasaki disease in India: Increasing awareness or increased incidence?. Perspect Biol Med.

[17] Singh S, Bhattad S (2016). Kawasaki disease incidence at Chandigarh, North India, during 2009–2014. Rheumatol Int.

[18] Bhat M (2016). Kawasaki disease in Jammu and Kashmir. Indian Pediatr.

[19] De Zorzi A, Colan SD, Gauvreau K, Baker AL, Sundel RP, Newburger JW (1998). Coronary artery dimensions may be misclassified as normal in Kawasaki disease. J Pediatr.

[20] Dietz SM, Tacke CE, Kuipers IM (2015). Cardiovascular imaging in children and adults following Kawasaki disease. Insights Imaging.

[21] Han BK, Lesser A, Rosenthal K, Dummer K, Grant K, Newell M (2014). Coronary computed tomographic angiographic findings in patients with Kawasaki disease. Am J Cardiol.

[22] Tsujii N, Tsuda E, Kanzaki S, Kurosaki K (2016). Measurements of coronary artery aneurysms due to kawasaki disease by dual-source computed tomography (DSCT). Pediatr Cardiol.

[23] Maji B, Banerjee S, Pal P (2014). Nephrotic syndrome in Kawasaki disease. Clin Pediatr (Phila).

[24] Khubchandani RP, Dhanrajani A (2014). Facial nerve palsy complicating a case of Kawasaki disease. Indian J Pediatr.

[25] Thapa R, Chakrabartty S (2009). Atypical Kawasaki disease with remarkable paucity of signs and symptoms. Rheumatol Int.

[26] Watanabe T (2015). Pyuria in patients with Kawasaki disease. World J Clin Pediatr.

[27] Vignesh P, Bhattad S, Singhal M, Singh S (2016). A 5-year-old boy with only fever and giant coronary aneurysms: The enigma of Kawasaki disease?. Rheumatol Int.

[28] Singh S, Agarwal S, Bhattad S (2016). Kawasaki disease in infants below 6 months: A clinical conundrum?. Int J Rheum Dis.

[29] Ha K-S, Jang G, Lee J (2013). Incomplete clinical manifestation as a risk factor for coronary artery abnormalities in Kawasaki disease: A meta-analysis. Eur J Pediatr.

[30] Kumar A, Singh S (2016). BCG site reactivation in Kawasaki disease. Arthritis Rheumatol Hoboken NJ.

[31] McCrindle BW, Rowley AH, Newburger JW, Burns JC, Bolger AF, Gewitz M, Baker AL, Jackson MA, Takahashi M, Shah PB, Kobayashi T, Wu MH, Saji TT, American Heart Association Rheumatic Fever, Endocarditis, and Kawasaki Disease Committee of the Council on Cardiovascular Disease in the Young, Council on Cardiovascular and Stroke Nursing, Council on Cardiovascular Surgery and Anesthesia, Council on Epidemiology and Prevention (2017). Circulation.

[32] Watanabe T, Abe Y, Sato S, Uehara Y, Ikeno K, Abe T (2007). Sterile pyuria in patients with Kawasaki disease originates from both the urethra and the kidney. Pediatr Nephrol Berl Ger.

[33] Reddy M, Singh S, Rawat A, Sharma A, Suri D, Rohit MK (2016). Pro-brain natriuretic peptide (ProBNP) levels in North Indian children with Kawasaki disease. Rheumatol Int.

[34] Dionne A, Meloche-Dumas L, Desjardins L (2016). NT-proBNP diagnostic algorithm for Kawasaki Disease compared to AHA Algorithm. Pediatr Int Off J Jpn Pediatr Soc.

[35] Bhagwat A, Mukhedkar S, Ekbote S, Gordon JB (2015). Missed Kawasaki disease in childhood presenting as myocardial infarction in adults. Indian Heart J.

[36] Rizk SRY, El Said G, Daniels LB (2015). Acute myocardial ischemia in adults secondary to missed Kawasaki disease in childhood. Am J Cardiol.

[37] Newburger JW, Takahashi M, Burns JC (2016). Kawasaki disease. J Am Coll Cardiol.

[38] Bhagwat A, Mukhedkar S, Ekbote S, Gordon JB (2015). Missed Kawasaki disease in childhood presenting as myocardial infarction in adults. Indian Heart J.

[39] Singh S, Bhattad S, Gupta A, Suri D, Rawat A, Rohit M (2016). Mortality in children with Kawasaki disease: 20 years of experience from a tertiary care centre in North India. Clin Exp Rheumatol.

[40] Chang R-KR (2002). Hospitalizations for Kawasaki disease among children in the United States, 1988–1997. Pediatrics.

[41] Huang S-K, Lin M-T, Chen H-C, Huang S-C, Wu M-H (2013). Epidemiology of Kawasaki disease: Prevalence from national database and future trends projection by system dynamics modeling. J Pediatr.

